# Progressive Multifocal Leukoencephalopathy after Treatment with Nivolumab

**DOI:** 10.3201/eid2408.180460

**Published:** 2018-08

**Authors:** Martin Martinot, Guido Ahle, Inesa Petrosyan, Camille Martinez, Dragos M. Gorun, Mahsa Mohseni-Zadeh, Samira Fafi-Kremer, Martine Tebacher-Alt

**Affiliations:** Hôpitaux Civils de Colmar, Colmar, France (M. Martinot, G. Ahle, I. Petrosyan, C. Martinez, D.M. Gorun, M. Mosheni-Zadeh);; Hôpitaux Universitaires de Strasbourg, Strasbourg, France (S. Fafi-Kremer, M. Tebacher-Alt)

**Keywords:** nivolumab, immune checkpoint inhibitor, progressive multifocal leukoencephalopathy, JC virus, viruses, meningtitis/encephalitis, HIV/AIDS and other retroviruses

## Abstract

Progressive multifocal leukoencephalopathy (PML) is increasingly being reported in patients undergoing immunotherapy. We report a case of progressive multifocal leukoencephalopathy after treatment with nivolumab, a PD-1 blocker that is used to restore impaired T-cell responses in patients with cancer and infections. Data for 4 other cases were obtained from pharmacovigilance databases.

Progressive multifocal leukoencephalopathy (PML) is a life-threatening disease of the central nervous system caused by JC polyoma virus (JCV). Although incidence of this disease reportedly has increased markedly in association with HIV/AIDS, a new complication caused by immune-mediated therapies, particularly monoclonal antibodies, has emerged (*1*).

Nivolumab, a cancer immunotherapy, is a checkpoint inhibitor that functions by blocking the programmed cell death 1 (PD-1)/programmed death ligand 1 pathway and restoring immunity against tumor cells (*2*). We report a case of PML that occurred after 12 months of treatment with nivolumab in a patient with refractory stage IV Hodgkin lymphoma.

A 54-year-old HIV-negative man was hospitalized in November 2017 for new-onset acute back pain that was not responsive to high-dose morphine. In 2013, he had been given a diagnosis of stage IV Hodgkin lymphoma, for which he underwent several chemotherapy sessions, including ABVD (adriamycin, bleomycin, vinblastine, dacarbazine); DHAP (dexamethasone, high-dose cytarabin, cisplatin); ICE (ifosfamide, carboplatin, etoposide); ChIVPP (chlorambucil, vinblastine, procarbazine, prednisolone); etoposide, gemcitabine, liposomal doxorubicin plus methylprednisolone; and brentuximab plus bendamustin. In 2016, a positron emission tomography scan showed new locations of lymphoma (lymph nodes, spine, and lungs).

He was then given nivolumab (3 mg/kg every 2 wk) during October 2016–November 2017. He was also taking hydrocortisone (30 mg/d) for hypocorticism; 110 mg/d starting in March 2017). At admission, corticosteroid therapy was increased to 40 mg/d of methylprednisolone to treat hyperalgesic spine locations.

Three weeks later, progressive hemiparesis developed on the left side of his body. Magnetic resonance imaging of the brain showed multiple nonenhancing lesions that were suggestive of PML (Figure). Analysis of cerebrospinal fluid showed identical concentrations of leukocytes and erythrocytes (1 cell/mm^3^). PCR analyses of showed a JCV concentration of 2,230 copies/mL in cerebrospinal fluid and 9,720 copies/mL in blood, confirming the diagnosis of PML.

**Figure:**
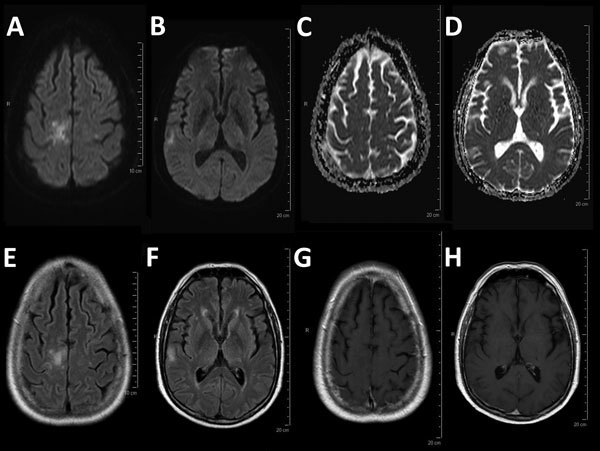
Magnetic resonance imaging for a 54-year-old man with progressive multifocal leukoencephalopathy after treatment with nivolumab, showing typical multifocal lesions: diffusion weighted imaging hyperintensity (A, B) with no decrease in the apparent diffusion coefficient (C, D), corresponding patchy corticosubcortical hyperintensities on fluid-attenuated inversion recovery image (E, F) without enhancement on T1-weighted imaging after administration of gadolinium (G, H).

Nivolumab was discontinued, and corticosteroid dose was decreased. CD4 lymphocyte subpopulation counts were 227 cells/mm^3^ (12%) and CD8 lymphocyte subpopulation counts 1,547 cells/mm^3^ (83%). The patient was still alive 5 months later but showed no neurologic improvement and had persistent JCV viremia (2,506 copies/mL).

PD-1 is a transmembrane receptor expressed on dendritic, NK, CD4, CD8, and T-cells and involved in downregulation of the immune system by promotion of activated T-cell apoptosis and diminution of regulatory T-cell apoptosis to stop unrestrained cytotoxic functions (*3*,*4*). Blockade of pathways involving PD-1 and its ligand is a promising treatment for cancers (melanoma, nonsmall cell lung cancer, metastatic renal cell carcinoma, head and neck carcinomas, Hodgkin lymphoma, and urothelial carcinoma) (*4*), although the role of PD1/programmed death ligand 1 in immune suppression and the mechanism of action of antibodies remain to be better defined.

Checkpoint inhibitors can cause unique toxicities, known as immune-related adverse events (IRAEs), including rashes, colitis, hepatitis, nephritis, pneumonitis, and thyroid disturbance, but these rarely comprise neurologic manifestations (<1%) such as aseptic meningitis and immune encephalitis (*3*,*5*,*6*). Neurologic IRAEs occur rapidly after treatment with checkpoint inhibitors and disappear after treatment interruption and introduction of steroids (*6*). Therefore, we excluded a diagnosis of neurologic IRAEs for this patient. Infections related to treatment with checkpoint inhibitors are infrequent (*4*).

Checkpoint inhibitors have been studied as treatment for chronic infectious diseases by restoring T-cell depletion (*2*,*3*,*7*), including PML, in which PD-1 on CD4 and CD8 cells has been reported (*1*,*8*). In a series of 740 patients given checkpoint inhibitors, serious infections developed in 7.4%, particularly in patients taking ipilimumab and nivolumab, steroids, or tumor necrosis factor antibodies for treatment of IRAEs, but few opportunistic infections and no PML were reported (*9*).

We found 4 unpublished cases of PML related to nivolumab in pharmacovigilance databases, but few data are available for these cases. One case of PML in the European database EudraVigilance did not contain any detailed information. We found 3 cases in the World Health Organization pharmacovigilance database: a 70-year-old man with metastatic melanoma who was treated with ipilimumab for 3 months and nivolumab for 9 months; a 61-year-old woman with lung cancer who was given nivolumab (300 mg) 21 days before PML developed; and a 61-year-old woman who was treated with nivolumab (300 mg every 15 d for 5 mo).

Another reported mechanism of development of infections with use of checkpoint inhibitors is restoration of T-cell function that mimics immune restoration syndrome (IRIS), which can cause reactivation of latent tuberculosis (*4*) and acute progression of aspergillosis. IRIS has been reported in patients with PML, but for our patient, use of steroids and onset of PML long after treatment with nivolumab was started did not support a diagnosis of IRIS.

This patient was highly immunocompromised because of lymphoma and use of steroids. Thus, it is difficult to determine whether nivolumab contributed to development of PML or whether PML was caused by combinations of different factors. PML cases have been reported even long after cure of the initial condition, but the incidence of PML among patients with hematologic malignancies is rare (0.07%) (*10*). However, because of the complexity and unknown mechanisms of checkpoint inhibitors and reported cases of PML, physicians should be vigilant when using this treatment.
